# traveltime: an R package to calculate travel time across a landscape from user-specified locations

**DOI:** 10.12688/gatesopenres.16356.1

**Published:** 2025-07-24

**Authors:** Gerard E. Ryan, Nicholas Tierney, Nick Golding, Daniel J. Weiss

**Affiliations:** 1 The Kids Research Institute Australia, Nedlands, WA, 6009, Australia; 2 The University of Melbourne School of Population and Global Health, Melbourne, Victoria, 3010, Australia; 3 Curtin University, Bentley, WA, Australia; 4 The University of Western Australia Department of Mathematics and Statistics, Perth, Western Australia, Australia

**Keywords:** R, geographic information systems, spatial analysis

## Abstract

Understanding and mapping the time to travel among locations is useful for many activities from urban planning to public health and myriad others. Here we present a software package — traveltime — written in and for the language R. traveltime enables a user to create a raster map of the travel time over an area of interest from a user-specified set of locations defined by geographic coordinates. The result is a raster of the area of interest where the value in each cell is the lowest travel time in minutes to the nearest of the supplied locations. We envisage this software having diverse applications including: estimating sampling bias, allocating defibrillators, setting health districts, or mapping access to vehicle chargers and agricultural facilities. The work-flow requires two key steps: preparing a friction surface for the area of interest, and then calculating travel time over that surface for the points of interest. traveltime is available from R-Universe and GitHub, and documented at https://idem-lab.github.io/traveltime/.

## Introduction

Understanding and mapping the time to travel among locations is useful for many activities from urban planning ( Zahavi 1974) to public health ( Hulland et al. 2019; Weiss et al. 2020) and myriad others ( Nelson et al. 2019). Global maps of travel time to cities ( Weiss et al. 2018; Nelson et al. 2019) and health care ( Hulland et al. 2019; Weiss et al. 2020) have generated much interest and use ^1^, and the city data set of Nelson et al. (2019) is available to R users through the widely-used geodata package ( Hijmans et al. 2024). Here we extend that work to enable travel time calculations from any arbitrary set of locations and friction surface.

We present a software package — traveltime — written in and for the language R ( R Core Team 2024). traveltime enables a user to create a raster map of the travel time over an area of interest from a user-specified set of locations defined by geographic coordinates. The result is a raster of the area of interest where the value in each cell is the lowest travel time in minutes to the nearest of the supplied locations.

A gaggle of R packages provide superficially similar though fundamentally different functionality via the TravelTime.com API ( TravelTime 2024a, 2024b; von Bergmann 2024; Lo Russo 2024). Their ‘Isochron’ polygons — areas reachable within a given time from a given location — are most comparable to what traveltime::calculate_travel_time() calculates. However, each isochron is a single polygon calculated is for a single point location and specified maximum travel time, rather than a raster of temporal gradation across a landscape, jointly for an arbitrary number of points, as in traveltime. TravelTime.com cannot provide a single result surface for time to the nearest of a group of points, and continuous time scale without extensive repeated iteration for all combinations of time and points, plus additional calculation of the minimum value for each cell from all points. Furthermore, TravelTime.com requires access keys, a paid subscription beyond a short free period, and caps queries, which add considerable friction to the use of this resource.

With traveltime, we provide free and open source software to estimate travel time from any number of user-supplied locations, across a complete area of interest, and with convenient access to motorised transport or walking friction surfaces with global coverage.

## Methods

### Implementation

traveltime is an R ( R Core Team 2024) package and requires installation of R version 4.1 or a more recent version traveltime provides a spatial interface using object classes from the terra package ( Hijmans 2024). Travel time is calculated as movement over a resistance ‘friction surface’ ( van Etten 2017). To provide easy access to the existing friction surfaces generated by Weiss et al. (2020), traveltime internally uses the R package malariaAtlas ( Pfeffer et al. 2018) to download surfaces for the area of interest; though users can also supply any other friction surface raster.

### Operation

The work-flow requires two key steps:

• preparing a friction surface for the area of interest, and then
• calculating travel time over that surface for the points of interest.

### Installation

traveltime is available from R-Universe and GitHub, and documented at https://idem-lab.github.io/traveltime/. It can be installed in R as follows:

install.packages("traveltime", repos = c("https://idem-lab.r-universe.dev"))


### Example: walking from public transport in Singapore

Here we provide an example to calculate and map the walking travel time from rail transport stations across Singapore. Complete code for this example is available as a vignettte in package documentation.

*Prepare data and friction surface*

We need two items of data:

• our area of interest — Singapore, and
• our points to calculate travel time from — Singapore’s Mass Rapid Transit (MRT) and Light Rail Transit (LRT) stations.

We download singapore_shapefile, a polygon boundary of Singapore from the GADM ( GADM 2022) database using the geodata package ( Hijmans et al. 2024) as our area of interest:

library(terra)
library(geodata)

singapore_shapefile <- gadm(
  country = "Singapore",
  level = 0,
  path = tempdir(),
  resolution = 2
)

singapore_shapefile 

 class       : SpatVector
 geometry    : polygons
 dimensions  : 1, 2  (geometries, attributes)
 extent      : 103.6091, 104.0858, 1.1664, 1.4714 (xmin, xmax, ymin, ymax)
 coord. ref. : lon/lat WGS 84 (EPSG:4326)
 names       : GID_0   COUNTRY
 type        : <chr>     <chr>
 values      :   SGP Singapore


Next we use the function traveltime::get_friction_surface to retrieve a walking friction surface for our area of interest. Alternatively, we could use surface = "motor2020" for motorised travel. We’re also only interested in walking *on land* so we mask out areas outside of the land boundary in singapore_shapefile. Users supplying their own friction surfaces do not need to download one in this fashion, only ensure that it is in SpatRaster format.

friction_singapore <- traveltime::get_friction_surface(
    surface = "walk2020",
    extent = singapore_shapefile
  )|>
  terra::mask(singapore_shapefile)

<GMLEnvelope>
    |-- lowerCorner: 1.1664 103.6091
    |-- upperCorner: 1.4714 104.0858Start tag expected, '<' not found

friction_singapore

class       : SpatRaster
dimensions  : 37, 57, 1 (nrow, ncol, nlyr)
resolution  : 0.008333333, 0.008333333 (x, y)
extent      : 103.6083, 104.0833, 1.166667, 1.475 (xmin, xmax, ymin, ymax)
coord. ref. : lon/lat WGS 84 (EPSG:4326)
source(s)   : memory
varname     : Accessibility__202001_Global_Walking_Only_Friction_Surface_1.1664,103.6091,1.4714,104.0858
name        : friction_surface
min value   :       0.01200000
max value   :       0.06192715


Our points are the traveltime::stations data, containing coordinates of all LRT and MRT station exits in Singapore ( Land Transport Authority 2019):

library(traveltime)
head(stations)

            x        y
[1,] 103.9091 1.334922
[2,] 103.9335 1.336555
[3,] 103.8493 1.297699
[4,] 103.8508 1.299195
[5,] 103.9094 1.335311
[6,] 103.9389 1.344999


We plot these data below. traveltime takes resistance values of friction ( van Etten 2017), so higher values of friction indicate more time travelling across a given cell.

*Calculate and plot the travel time*

With all the data collected, the function calculate_travel_time() takes the friction surface friction_singapore and the points of interest in stations, and returns a SpatRaster of walking time in minutes to each cell from the nearest station:

travel_time_singapore <- calculate_travel_time(
  friction_surface = friction_singapore,
  points = stations
)

travel_time_singapore

class       : SpatRaster
dimensions  : 37, 57, 1 (nrow, ncol, nlyr)
resolution  : 0.008333333, 0.008333333 (x, y)
extent      : 103.6083, 104.0833, 1.166667, 1.475 (xmin, xmax, ymin, ymax)
coord. ref. :  
source(s)   : memory
name        : travel_time
min value   :           0
max value   :         Inf


**Figure 1.**
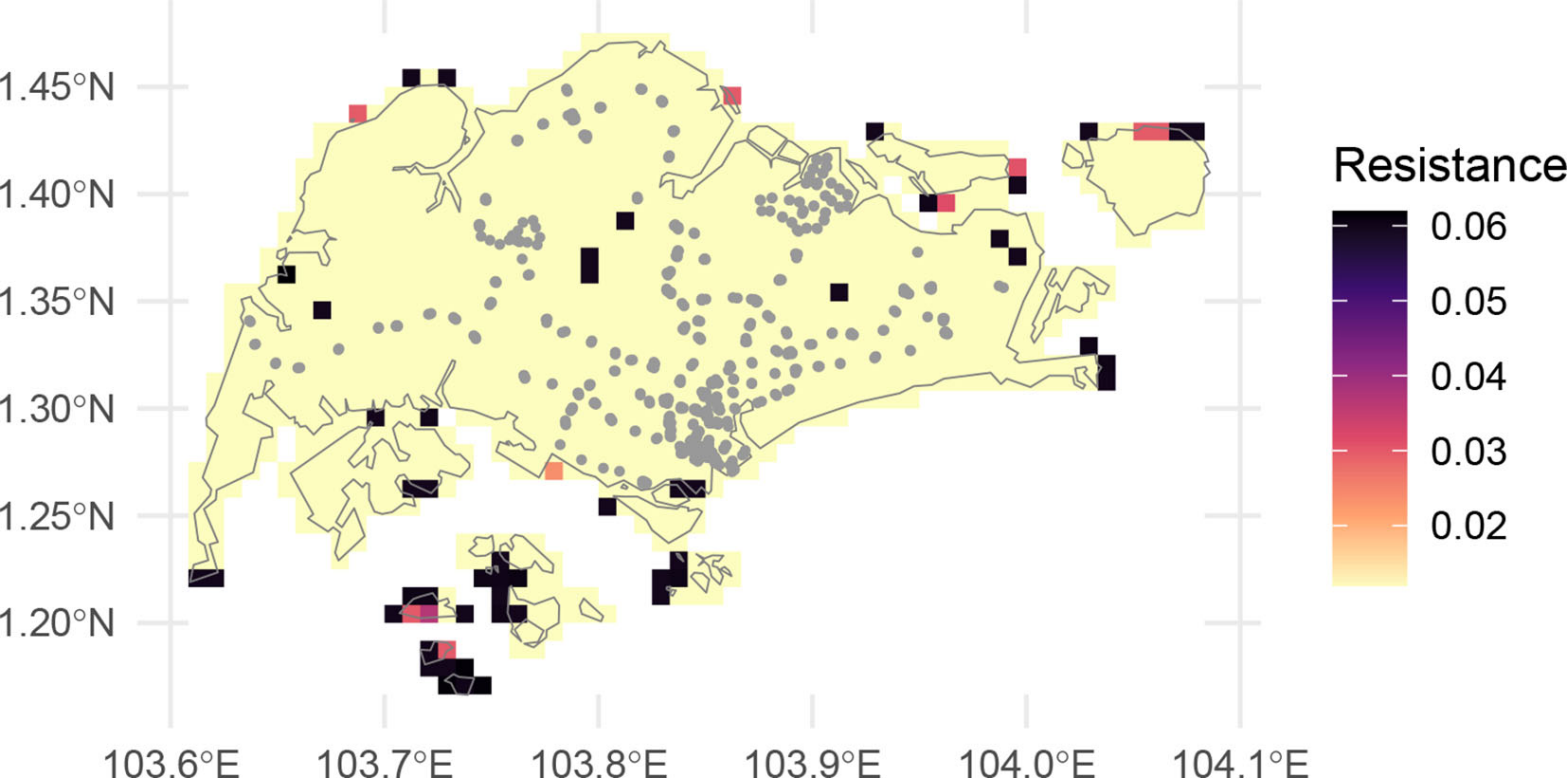
Friction surface raster of Singapore, showing Singapore boundary in grey, and station locations as grey points.

**Figure 2.**
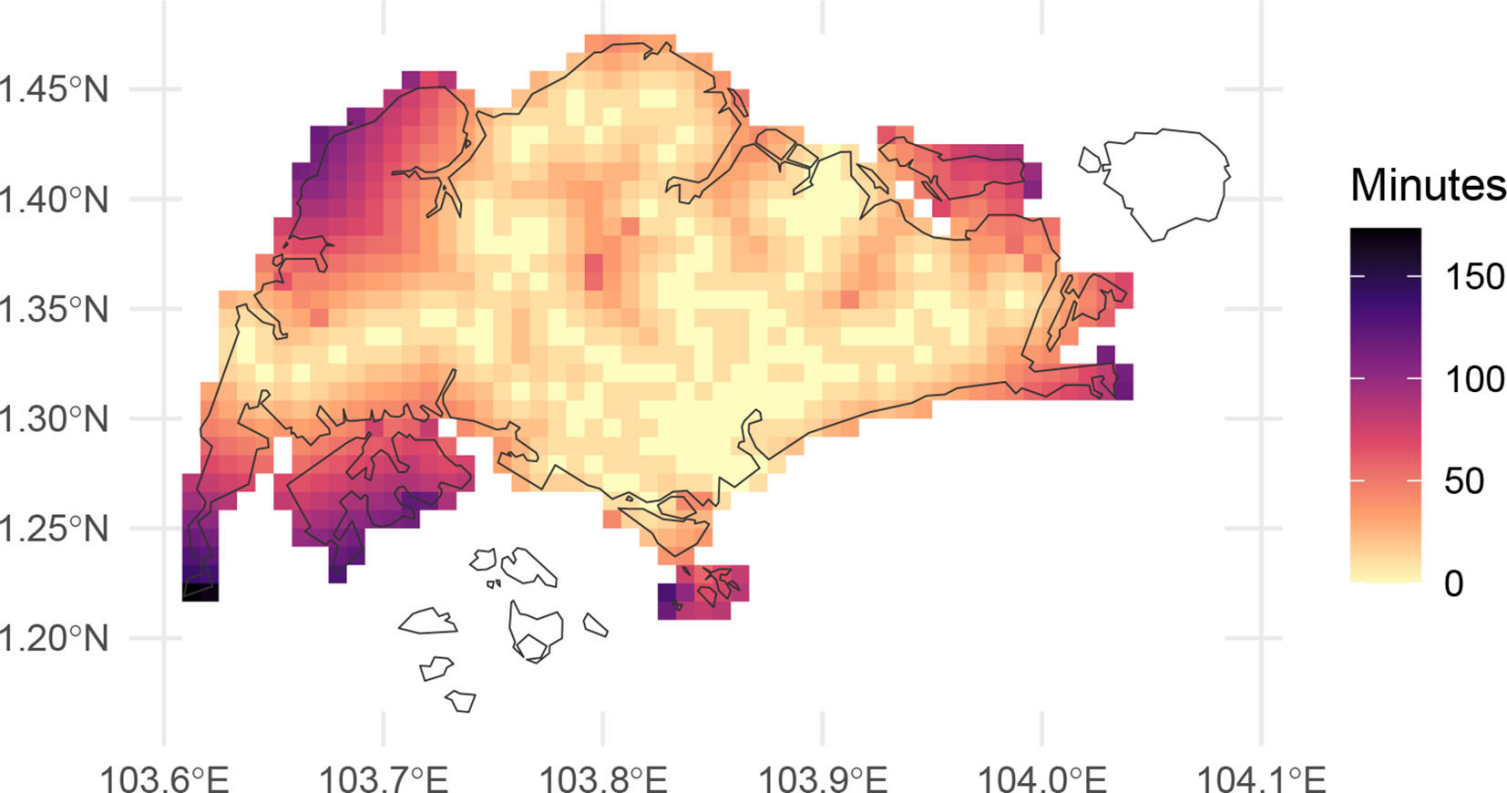
Map of walking travel time in Singapore, in minutes from nearest MRT or LRT station.

We present the resulting calculated travel times in Figure 2 where, as expected, the travel times are lowest near station exits (per Figure 1) and progressively higher further away. Note that the results in travel_time_singapore include infinite values ( Inf above). In Figure 1, the islands to the south and north-east are shown as filled cells, but unconnected with the mainland. The raster cells for these islands appear absent in Figure 2. Because they are not connected to any cells with a station, the calculated travel time is infinite, and so these cells do not appear in Figure 2.

## Discussion

The traveltime package is immediately suitable to be used ‘out-of-the-box’ with any set of coordinates, in any part of the globe. We envisage this software having diverse applications including: estimating sampling bias ( Dennis and Thomas 2000), allocating defibrillators ( Tierney et al. 2018), setting health districts ( Padgham et al. 2019), or mapping access to vehicle chargers ( Falchetta and Noussan 2021) and agricultural facilities ( Zhao et al. 2023). Nonetheless, we see opportunities to build the package utility through:

• capability to better distribute a wider range friction surfaces, and
• additional methods to efficiently compute results over large spatial extents.

Firstly, traveltime currently facilitates access to walking and motorised friction surfaces for 2020, both at 30 arc-second resolution (approximately 0.008333 decimal degrees, or just below 1 km ${\;}^{2}$ at the equator). Although the user can presently supply their own friction surface, we expect most applications will use these existing surfaces given the extensive work needed in creating new ones ( Weiss et al. 2018, 2020). As landscapes are dynamic, it may be useful to incorporate updated versions of these friction surfaces if and when they are available. Furthermore, although the resolution of these data is likely to be suitable for larger landscape foci, higher resolution data may be helpful for more locally focussed analyses. For instance, although the example here was chosen for it’s simplicity and low computational demands, a ~1 km ^2^ cell size is a relatively large area to walk across, and thus actual waking times may vary significantly within each cell. We underline however that a user can provide their own higher resolution friction surface at present if desired.

At the other end of the scale, as the area of interest increases, the size of the matrix of calculations necessary increases exponentially, making significant memory demands for analyses over large landscapes (e.g. analyses over Africa required ~ 72 GB RAM to run successfully). Developing methods to handle large landscapes either with less memory or via cloud resources would be helpful to make such analyses accessible to those without access to larger computing resources.

Lastly, although this article is intended to be the key reference for the traveltime package, we suggest citations of the package are accompanied by citing the underlying methodological work as well ( Weiss et al. 2018, 2020).

## Figure permissions

The authors confirm ownership of the figures used in this manuscript.

## Data and software availability

All software described here is available from R-Universe https://idem-lab.r-universe.dev/traveltime and GitHub https://github.com/idem-lab/traveltime, and documented at https://idem-lab.github.io/traveltime/. Code used in this paper is released via GitHub as v4, and archived on Zenodo under DOI 10.5281/zenodo.15347016.

The package associated with this paper contains information from the dataset “LTA MRT Station Exit (GEOJSON)” accessed on the 10th of December 2024 from data.gov.sg, which is made available under the terms of the Singapore Open Data Licence version 1.0 https://data.gov.sg/open-data-licence.

## Footnotes

^1^
Collectively >1600 citations per Google Scholar at the 28th of January 2025.
